# Supplementary material for: Comparing chromatin contact maps at scale: methods and insights
Source: Nat Methods. 2025 Mar 19;22(4):824–33. doi: 10.1038/s41592-025-02630-5 (PMC11978506; doi:10.1038/s41592-025-02630-5)
Supplement: Supplementary file 1 — Supplementary Figures 1–10, Supplementary Note, and Supplementary References. [file 41592_2025_2630_MOESM1_ESM.pdf]

nature methods

Supplementary information https://doi.org/10.1038/s41592-025-02630-5

Comparing chromatin contact maps at scale: methods and insights

In the format provided by the authors and unedited

Supplementary Figures

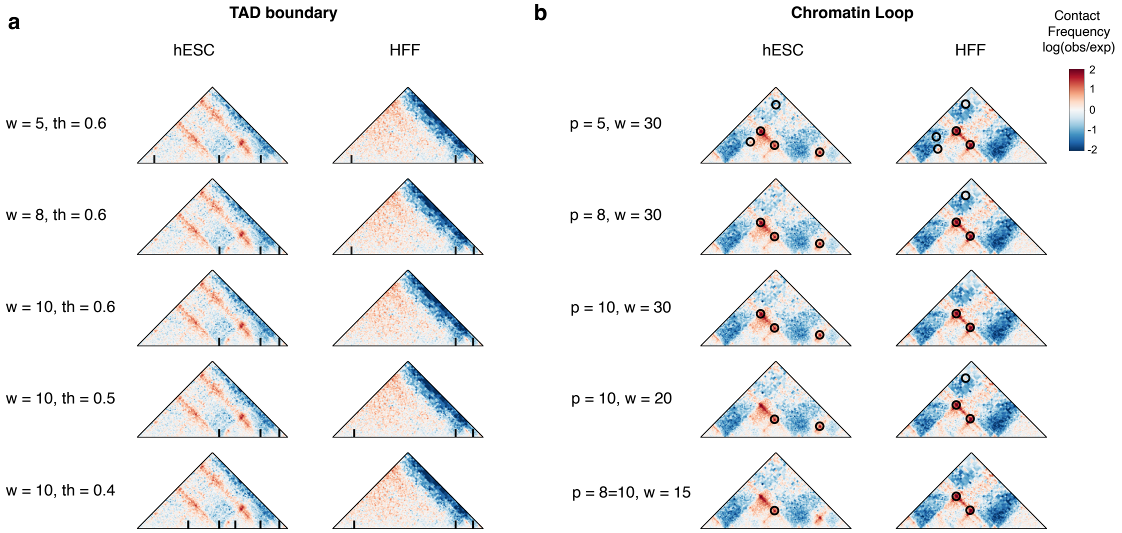

Supplementary Figure 1. Sensitivity of TAD and loop caller on parameter shifts. a, TAD boundaries (highlighted with black bar) called with different sizes of diamond-shaped window (*w*) and thresholds of insulation scores (*th*). **b,** Chromatin loops (highlighted with black circle) identified using different sizes of center window (*p*) and donut filter (*w*). Example maps used here are the same as in **Extended Data Fig. 2b**.

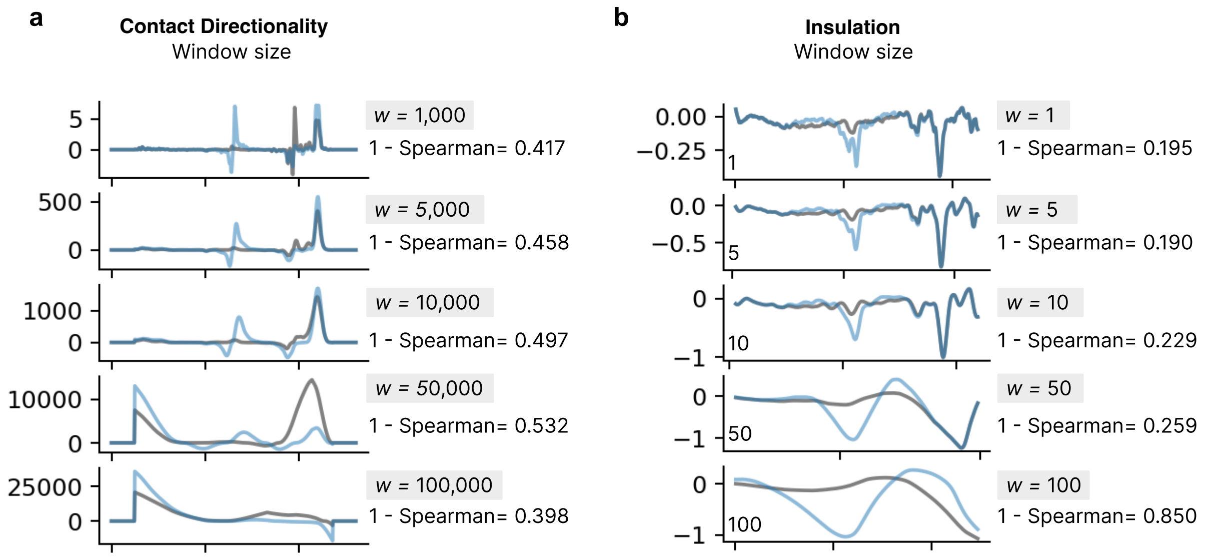

Supplementary Figure 2. Sensitivity of directionality index and insulation tracks on parameter shifts. Contact Directionality (**a**) and Insulation (**b**) tracks across a range of input window size choices, w, as well as the resulting Spearman's correlation between the two tracks. A window size of 1 Mb was used for both approaches to produce the *in silico* scoring results in the **Results** section.

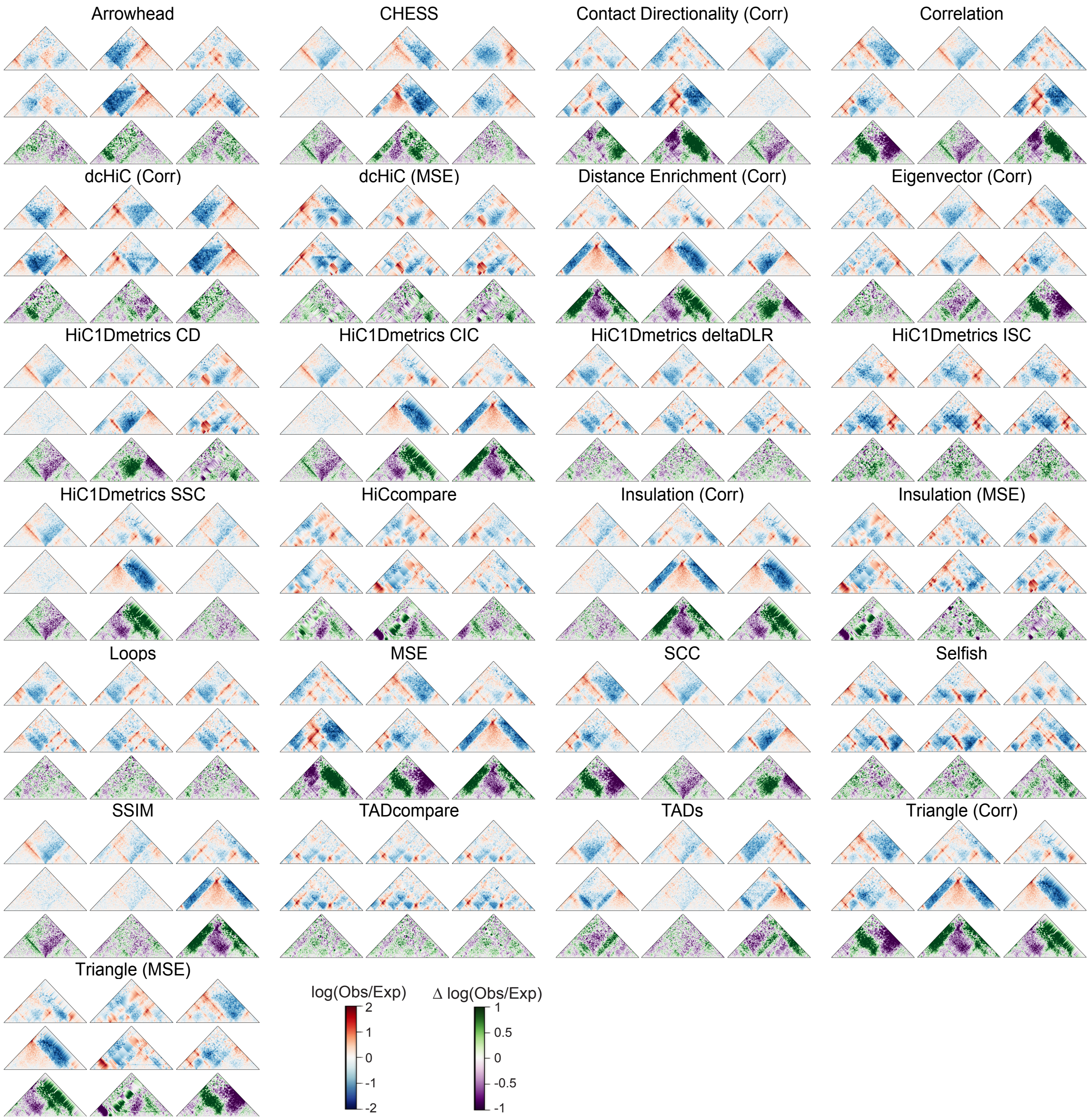

Supplementary Figure 3. The three most disruptive experimental map pairs of each scoring method. For each example row, the MicroC ESC map is shown on the top, the MicroC HFF map is shown in the middle, and the difference between the two maps is shown on the bottom. The top three disruptive maps were chosen for the Micro-C 1 Mb DEG windows in chromosomes 21 and 22.

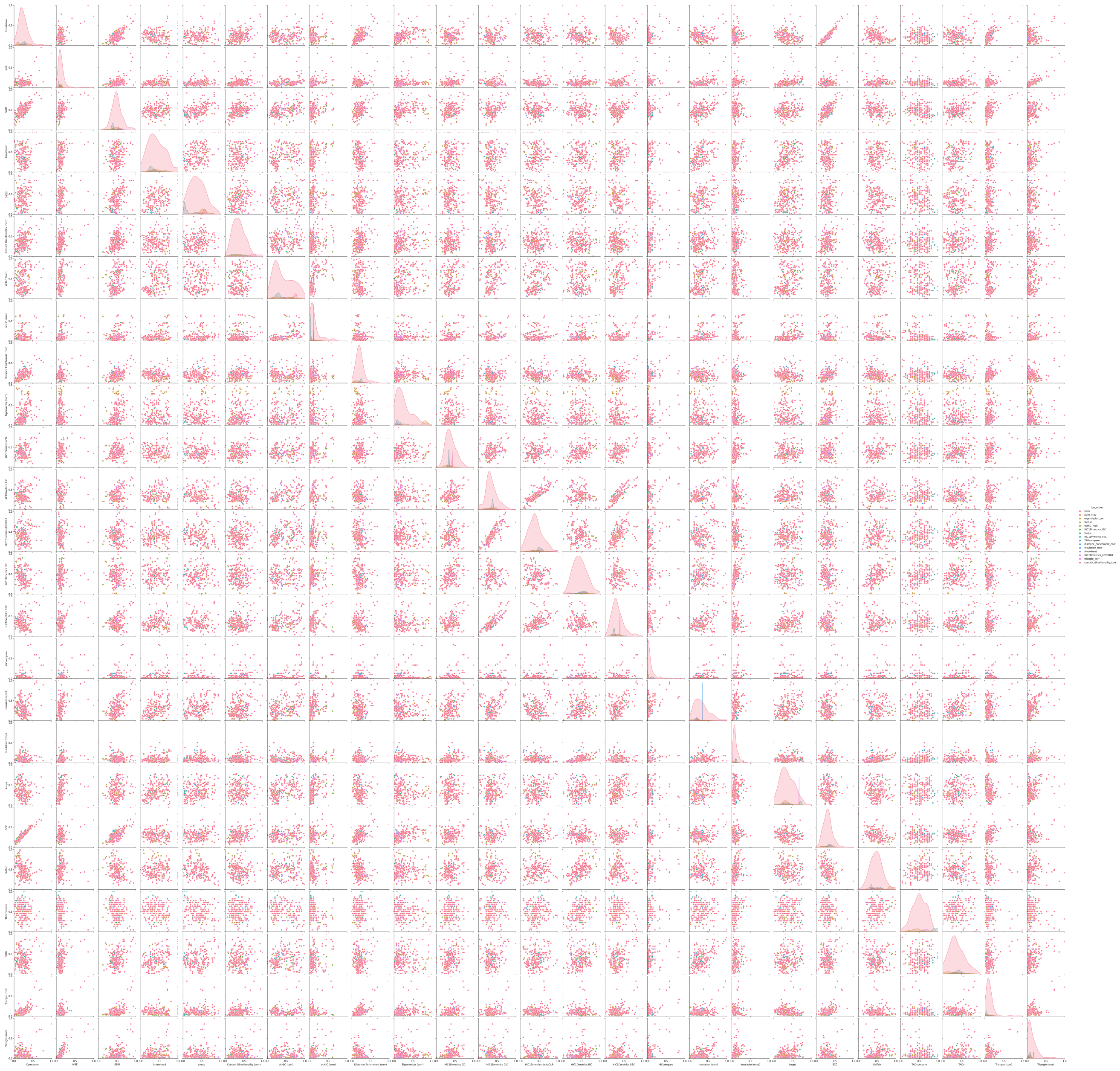

Supplementary Figure 4. Pairwise correlation across methods. DEG window scores for 1 Mb windows between MicroC ESC and HFF are plotted across methods (rows and columns). Colors represent windows that are uniquely scored in the top 5% by only one method and pink dots represent the rest of the windows.

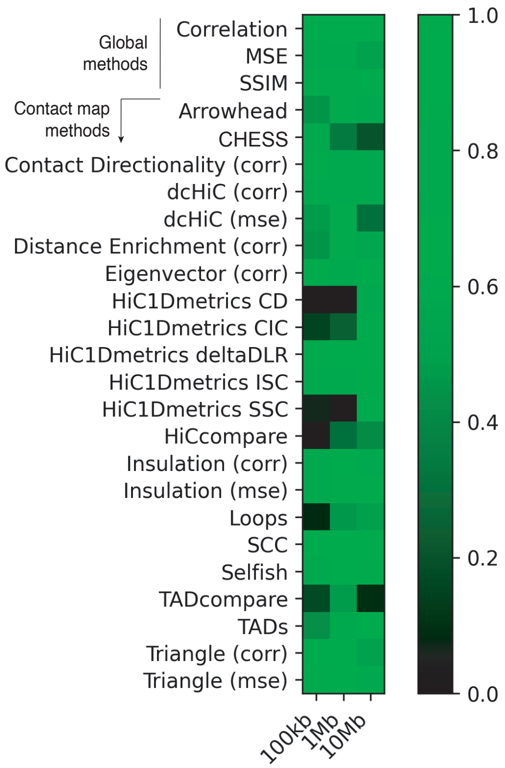

Supplementary Figure 5. Comparing scores between Micro-C and Hi-C data. Scores for DEG regions at 100 kb, 1 Mb, and 10 Mb windows are compared between Micro-C and Hi-C data using Spearman's correlation (green color scale). Some methods show high correlation between experiment types at all three window sizes, such as Correlation, SSIM, Contact Directionality (corr), Eigenvector (corr), HiC1Dmetrics deltaDLR and ISC, Insulation (mse and corr), SCC, and Selfish. The rest of the methods show varying degrees of correlation across window sizes, although no methods show poor correlation between Micro-C and Hi-C data across all three window sizes. 100 kb and 1 Mb windows use a resolution of 2048 bp, while 10 Mb windows use a resolution of 20480 bp, so that the bin size is scaled up with the window size.

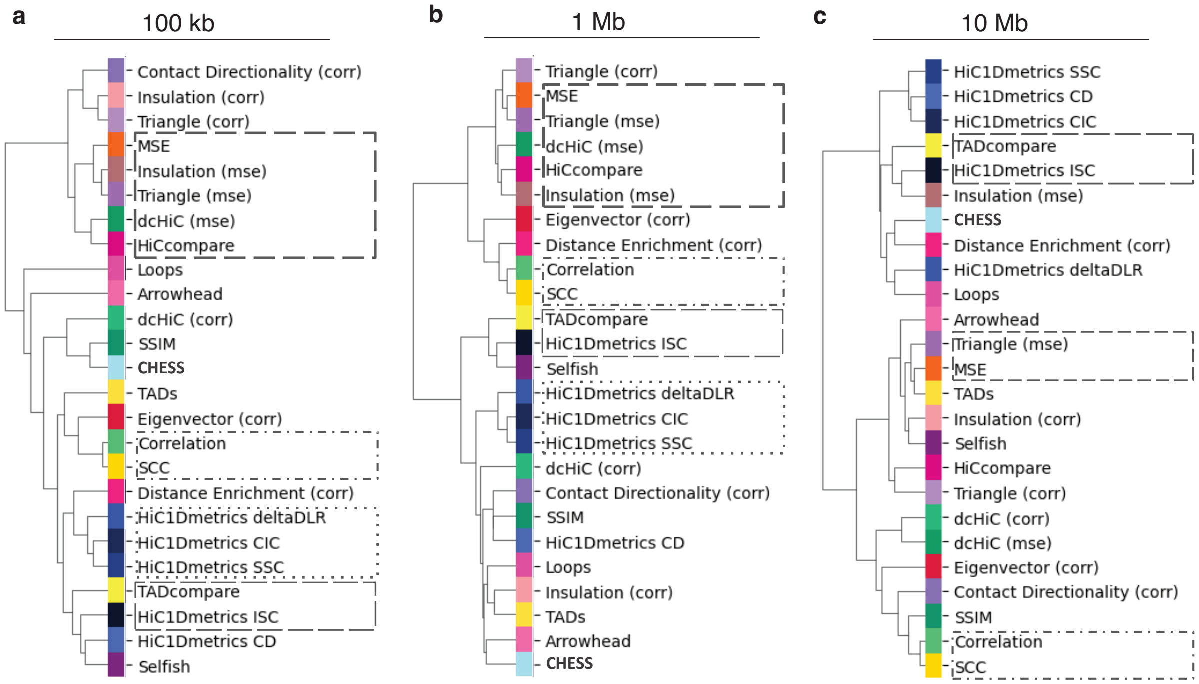

Supplementary Figure 6. Comparing method clustering across map sizes. DEG windows of size **a,** 100 kb, **b,.** 1 Mb, and **c,** 10 Mb compared between Micro-C ESC and HFF and the resulting scores clustered across 25 methods. Groups of methods that cluster together across window sizes are boxed in various types of dotted lines. One pair of methods clusters together across all three window sizes: TADcompare and HiC1Dmetrics ISC. Others group together in only two of the three window sizes such as HiC1Dmetrics deltaDLR, CIC, and SSC, which cluster in 100 kb and 1 Mb windows but not 10 Mb windows. Similarly, Correlation and SCC clutter in 100 kb and 10 Mb, but not 1 Mb windows. Lastly, one cluster of 5 methods is matched between 100 kb and 1 Mb—MSE, Insulation (mse), Triangle (mse), dcHiC (mse), and HiCcompare—but only two of these—MSE and Triangle (mse)–– cluster together in 10 Mb windows. Overall, there are more similarities in clustering between 100 kb and 1 Mb maps than 10 Mb maps.

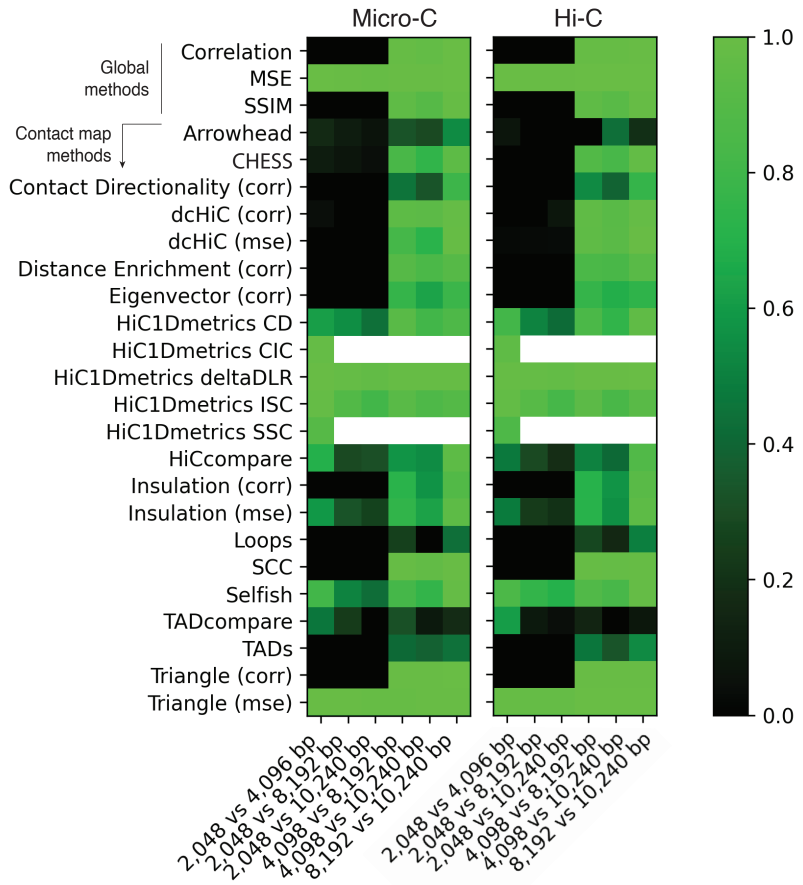

Supplementary Figure 7. Comparing disruption scores at different resolutions. DEG 1 Mb window scores are compared between maps with different resolutions, considering all possible pairs from the following: 2,048 bp, 4,096 bp, 8,192 bp, 10,240 bp, using data from Micro-C and Hi-C. Some methods show consistent results across different resolutions in both Micro-C and Hi-C data, while some are only consistent at certain resolutions. Many methods show consistent results (high correlation, more green) at all resolutions but the highest, 2,048 bp, suggesting that these methods are better performing for lower resolution maps. Additionally, some methods show varied results at all resolutions (low correlation, more black), such as Arrowhead, Loops, TADcompare, and TADs. This is expected since these methods call loops and TADs which are highly affected by resolution. Interestingly, Micro-C data shows more consistent results across resolutions overall (more green), which might be expected since the data is expected to perform better at higher resolutions.

a

b

c

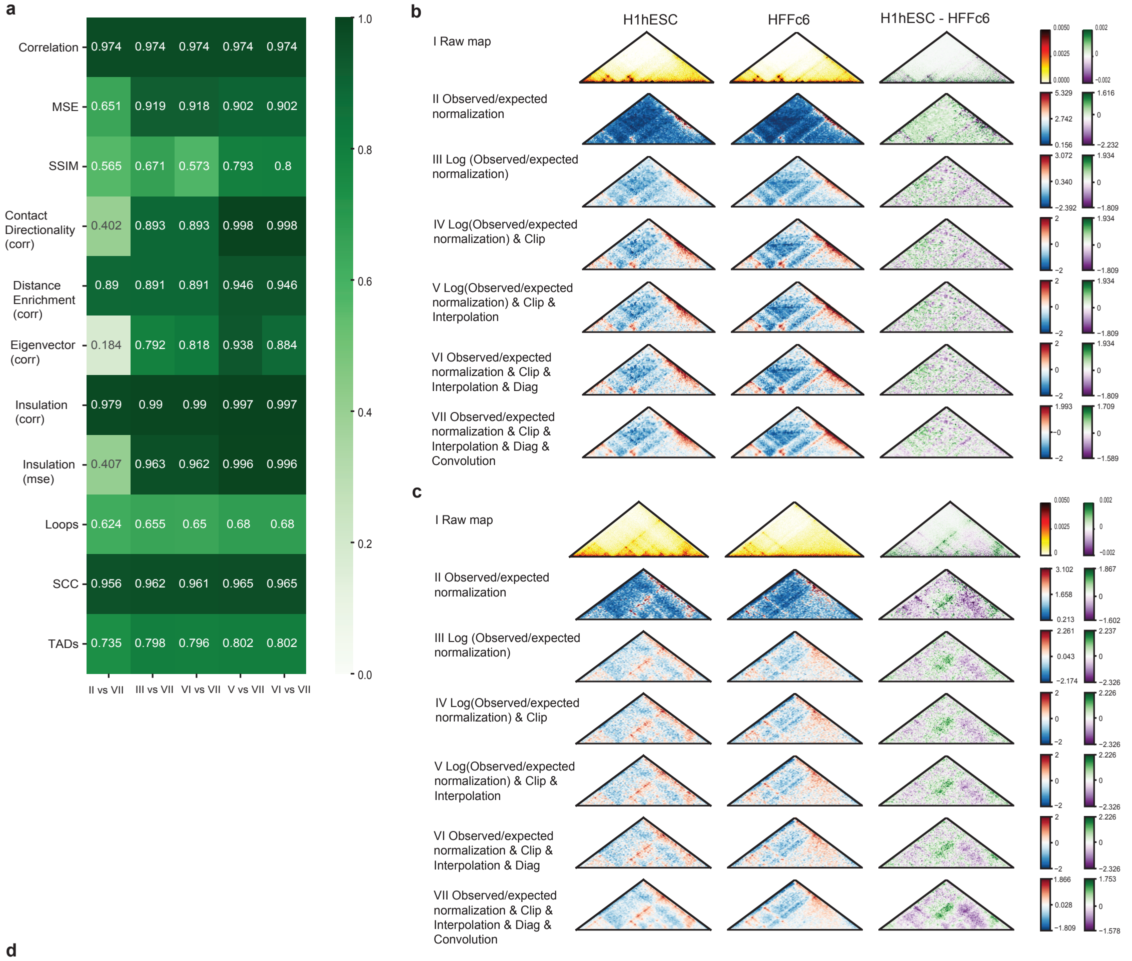

d

Normalization	Example	Correlation	MSE	SSIM	Contact Directionality (corr)	Distance Enrichment (corr)	Eigenvector (corr)	Insulation (corr)	Insulation (mse)	Loops	SCC	TADs
II	B	0.313	0.488	0.158	0.135	0.089	0.180	0.200	0.042	0.539	0.328	0.357
	C	0.719	0.420	0.481	0.588	0.580	0.847	0.730	0.056	0.513	0.718	0.458
III	B	0.313	0.270	0.825	0.170	0.089	0.068	0.196	0.034	0.543	0.341	0.462
	C	0.719	0.349	0.949	0.806	0.582	1.364	0.724	0.060	0.575	0.711	0.5
IV	B	0.313	0.268	0.873	0.168	0.089	0.068	0.196	0.034	0.515	0.341	0.462
	C	0.719	0.349	0.959	0.806	0.582	1.364	0.724	0.060	0.574	0.711	0.5
V	B	0.313	0.268	0.876	0.168	0.089	0.068	0.195	0.034	0.533	0.342	0.462
	C	0.720	0.349	0.961	0.806	0.582	1.364	0.726	0.060	0.577	0.712	0.5
VI	B	0.313	0.268	0.876	0.169	0.089	0.065	0.195	0.034	0.533	0.342	0.462
	C	0.720	0.349	0.960	0.803	0.588	1.362	0.726	0.060	0.577	0.712	0.5
VII	B	0.195	0.148	0.762	0.167	0.078	0.060	0.185	0.034	0.619	0.211	0.429
	C	0.622	0.197	0.905	0.827	0.554	1.353	0.722	0.059	0.632	0.596	0.667

Supplementary Figure 8. Effects of map preprocessing steps on scoring metrics. We broke our contact map data processing pipeline into six discrete steps, applied sequentially: Starting with (I) count matrices, (II) compute observed/expected values, (III) log transform, (IV) clip to (-2,2), (V) perform linear interpolation, (VI) set diagonal values to NaN, and (VII) apply convolution. To evaluate the effects of each step on scoring methods, we analyzed DEG windows on chromosomes 21 and 22 (H1hESC vs. HFFc6), stopping the processing after each step and applying the scoring methods to the partially processed data. **a,** The multi-step processing after observed/expected normalization has limited effects on the results of map comparison methods. The heatmap shows the Spearman's correlation for the scoring methods on maps with partial processing (II, III, IV, V or VI) versus full processing (VII). High correlations are observed between most comparisons except the one between log transformation (II) and all processing (VII). **b-d,** Two example maps that visually show a low (**b**) or high (**c**) level of contact differences between H1hESC and HFFc6 experimental maps illustrate the benefit of log transformation. **d,** MSE in particular benefits from the log transformation,

better capturing the visually notable differences in map in (**c**) and scoring the more subtle differences in map (**b**) lower after log transformation (compare row III to row II, highlighted in red).

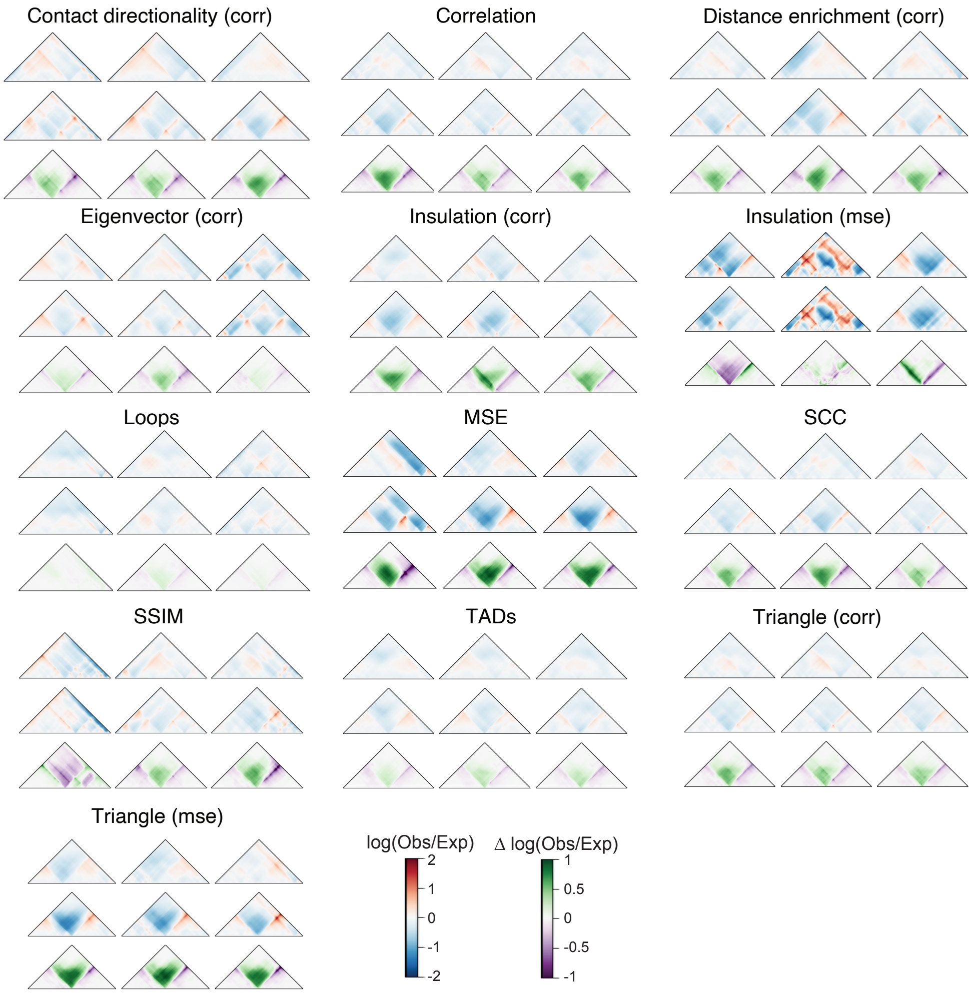

Supplementary Figure 9. The three most disruptive predicted map pairs of each scoring method. For each example row, the unperturbed map is shown on the top, the perturbed map is shown in the middle, and the difference between the two maps is shown on the bottom. The top three disruptive maps were chosen across the *in silico* screen.

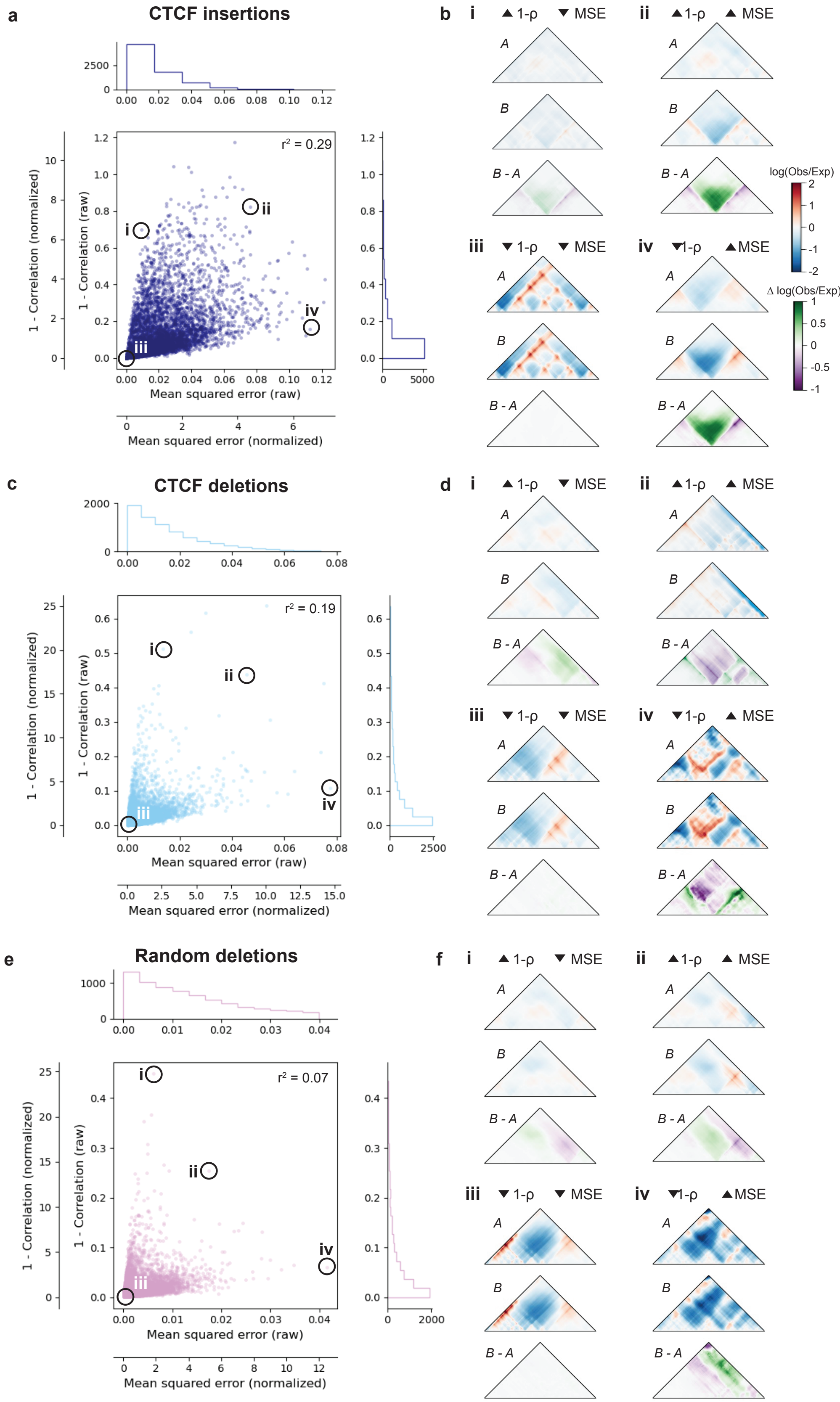

a
CTCF insertions
r² = 0.29
1 - Correlation (normalized)
1 - Correlation (raw)
Mean squared error (raw)
Mean squared error (normalized)
i
ii
iii
iv
b
▲1-ρ ▼MSE
▲1-ρ ▲MSE
▼1-ρ ▼MSE
▼1-ρ ▲MSE
A
B
B - A
log(Obs/Exp)
Δ log(Obs/Exp)
c
CTCF deletions
r² = 0.19
d
e
Random deletions
r² = 0.07
f

Supplementary Figure 10. Global methods to compare contact frequency maps rank maps differently on *in silico* perturbations. Mean squared error (MSE) vs Spearman's correlation (ρ) scores across an *in silico* screen of 7,500 map pairs with and without **a-b,** CTCF insertions, **c-d,** CTCF deletions, and **e-f,** random 100 bp deletions, similar to **Fig. 2.** We plot 1 - ρ such that higher values for both methods reflect increasing differences between maps. MSE versus Spearman's correlation are plotted where each point represents a comparison between a reference and perturbed map (**Fig. 4a**). Normalized scores are divided by the mean of the distribution of random deletions. Across all three perturbations, there is a weak relationship between the two disruption scores (r^2 = 0.29, 0.19, and 0.07 for CTCF insertions, CTCF deletions and random deletions, respectively). The relationship is strongest for CTCF insertions, for which scores are highest, followed by CTCF deletions, which have the next highest scores. Yet perturbations with at least one high score are not always concordantly scored. Examples of extreme scores for each perturbation are shown in B, D, and F in panels i through iv, illustrating that perturbations with high MSE and low 1 - ρ are consistently maps with high contrast, while low MSE and high 1 - ρ perturbations are maps with overall low contrast.

Supplementary Note

Method descriptions

Global methods

Correlation

The Spearman's rank correlation coefficient (ρ) assesses the correlation between the intensity of corresponding pixels in two maps by quantifying how well the relationship between the corresponding pixels can be described using a monotonic function. If the rank of intensity of all pixels in two contact maps are the same, the correlation is 1. If there is no relationship between the rank of pixel intensity between maps, the correlation is 0. Spearman's Rank Correlation coefficient can be described as:

$$\rho = 1 - \frac{6\Sigma d^2_i}{n(n^2-1)},$$

where the number of points in the data set is represented by n, and d^2 is the squared difference in the ranks of a single coordinate y_i between the two maps, which is summed over all points.

Correlation coefficients have been used extensively to compare contact maps[1–4]. Large-scale structural changes have high scores with correlation, because the ranks of each pixel in the maps are very different. This approach works well even when the contact intensity is low because magnitude of the values is not considered when converting to rank. However, Spearman's correlation is low even when the contact intensity is negligible (e.g. at an extreme, random noise will generate a very low correlation). The method does not pick up on small or focal changes in intensity, nor does it prioritize large-scale changes in intensity that do not change the map structure– the rank will stay the same even if the magnitude of the values change. Because matrices are flattened before calculating correlation, correlation also ignores the physical relationships between pixels of the map.

MSE

The mean squared error (MSE) measures the average squared difference between two flattened contact matrices, such that

$$\text{MSE} = \frac{1}{n} \sum_{i=1}^{n} (y_i - \tilde{y}_i)^2$$

Because MSE is a measure of absolute difference, it consistently prioritizes the greatest changes in intensity between contact maps. MSE has been widely adopted across machine learning as a loss function for consistent performance and ease of use [3–6] . Large changes between maps score highly, while visually smaller or localized changes produce lower MSE values. However, maps with differences in read count or normalization intensity will produce high MSE, despite little change in structure. For this reason, technical artifacts may dominate top map rankings scored by MSE. MSE will also deprioritize maps with large structural changes and low overall contact intensity. 2D map features will not be individually captured since the matrices are collapsed to 1D vectors.

SSIM

Structural similarity index measure (SSIM) quantifies the perceived change in structural information of two images by incorporating three terms:

Luminescence: Contrast: Structure:

$$l(x,y) = \frac{2\mu_x\mu_y + c_1}{\mu_x^2 + \mu_y^2 + c_1} \qquad c(x,y) = \frac{2\sigma_x\sigma_y + c_2}{\sigma_x^2 + \sigma_y^2 + c_2} \qquad s(x,y) = \frac{\sigma_{xy} + c_3}{\sigma_x\sigma_y + c_3}$$

Where the integrated SSIM score is equal to:

$$\text{SSIM}(x,y) = \left[l(x,y)^\alpha \cdot c(x,y)^\beta \cdot s(x,y)^\gamma\right]$$

SSIM is well-suited for identifying structural changes, and unlike correlation and MSE measures, is not biased by map contrast values. For this reason it has been incorporated into Hi-C map comparison methods previously[7]. However, SSIM is sometimes very sensitive to small changes relative to larger-scale changes that may appear more pronounced to the human eye. SSIM is also sensitive to the order of the input. It should be applied to the matrix as a whole (not vector-by-vector) as it is designed to account for neighboring values. NaN values must be interpolated or masked to zero.

Contact map methods

Arrowhead

Arrowhead[8] is a java-based algorithm designed to annotate contact domains from juicer .hic files. It achieves this by applying an “arrowhead” transformation, which converts the domain in the normalized contact matrix to a relatively-easy-to-annotate arrowhead-shaped feature in the arrowhead matrix. Dynamic programming is applied to the arrowhead matrix to identify the corners of domains. For our analysis, we first identified the contact domains at a chromosome-wide level for ESC and HFF Micro-C and Hi-C data at five different resolutions (2048 bp, 4096 bp, 8192 bp, 10240 bp, 20480 bp) and then subsetted the domains within each DEG window region on chr 21 and chr 22 by limiting both boundaries of the domains within the window. Chromosomes 21 and 22 were selected to be consistent with other methods. For the parameters, the sliding window size was set to 2000 and KR normalized was selected. The boundaries of the domains that are shared, gained or lost between ESC and HFF for each window were identified for comparison. The boundaries that are located within a certain distance (10,240 bp) are considered as shared.

CHESS

CHESS[7] measures the similarity between any pair of normalized chromatin contact matrices by calculating SSIM following observed/expected transformation using sklearn. The difference to the SSIM we implement is that they compare the calculated SSIM to a background to calculate a z-score and p-value. This indicates that the results are sensitive to the selection of background. Therefore, in our analyses, we only used the SSIM scores from CHESS before z-score normalization for comparison. The input formats of contact map that CHESS could take include fanc .hic, juicer .hic, cooler .cool or .mcool, and sparse contact matrix. We used .mcool files from ESC and HFF Micro-C and Hi-C data in our case. The comparison was made for DEG regions on chromosome 21 and 22 at five different resolutions, the same as Arrowhead.

Contact Directionality

Contact Directionality is inspired by Directionality Index (DI), which is a measure of contact frequency bias towards either upstream sequence or downstream sequence at each DNA locus. An inflection of DI values from negative to positive and vice versa indicates a potential chromatin boundary, where DI can be calculated by:

$$DI = \frac{B - A}{|B - A|} * \left(\frac{(A - E)^2}{E} + \frac{(B - E)^2}{E}\right)$$

Where A is the number of reads (or average normalized frequency value) that map from a given locus to upstream bins, B is that value for downstream bins, and E is the expectation under the null hypothesis, equal to $\frac{(A+B)}{2}$.

DI was first proposed by Dixon et al. in 2012[9]. It depends on two parameters: the size of the focal bin whose relative upstream and downstream contact frequency is being compared, and the size of the upstream and downstream bins (40 kb and 2 Mb in the original publication). To create a composite DI disruption score for a variant, DI is calculated for each locus in the region of both maps, creating a one-dimensional track that indicates whether the DNA at that locus mostly interacts with downstream genomic regions (positive DI values) or upstream (negative DI values). When these values are plotted along the x-axis of genomic coordinates (e.g. **Extended Data Fig. 2**), the location of potential chromatin boundaries are characterized by an inflection from strongly negative to strongly positive DI. To create the composite Contact directionality score, DI tracks are compared using MSE or correlation. This composite score is subject to the caveats of the chosen comparison method.

Contact Directionality parameters:
(1) size of the focal bin where upstream and downstream contact frequency is compared
(2) size of upstream and downstream bins

dcHiC
dcHiC[10] is a package for identifying significant compartment changes among two and more contact maps, including the ones that are not accompanied by a compartment flip. Specifically, it computes the compartment scores for each genomic bin by using singular value decomposition, applies quantile normalization of the scores across all samples, and then calculates the Mahalanobis distance from the normalized component scores, which is used to assign a statistical significance using the chi-square test. It accepts Hi-C pro style sparse matrix as its input. In our analyses, we first converted the .cool files from ESC and HFF Micro-C and Hi-C to the required sparse matrix and ran the comparison at a chromosome-wide level on chromosome 21 and 22 for the five different resolutions. We didn't use the resulting p-values for each compartment bin for the comparison with other methods. Instead, we extracted the quantile normalized compartment scores for each genomic bin in each DEG window for both ESC and HFF and calculated the MSE and correlation between the scores of ESC and HFF for each window.

Distance Enrichment
Distance Enrichment is inspired by contact decay, or the P(S) curve, which measures chromatin interaction as a function of genomic distance[11,12]. Interaction frequency across the contact map is ranked by genomic distance between all pairs of contact, resulting in a track of distance vs interaction frequency. That is, for each distance, all the possible bin pairs at that distance are summarized (mean or median). This results in a plot where distances 1-n bins are on the x-axis and the summary of contact frequency between all the bins at each distance is plotted. Decay curves may be calculated at a given resolution such that the chromosome is divided into n = L/r bins, where L is the chromosome length and r is resolution. Across an n x n contact map, the contact frequency of each entry $A_{i,j}$ is ordered by the distance between loci, i-j. Without observed over expected normalization, as distance increases, the probability of contact between loci decreases. A steeper decay in contact frequency indicates a greater distance between further loci, while a shallow contact decay suggests more interaction between distant loci. In the case of observed over expected normalization,

flattened decay is generally observed along the distances, with fluctuations representing changes of interaction frequency at the corresponding distances. Contact decay measures a global signal of relative interaction increase or decrease, but will not be sensitive to local structural changes to contact matrices.

Distance Enrichment parameters:
(1) mean vs median

Eigenvector
This method is inspired by genomic compartments, which are called by calculating the first eigenvector from Hi-C contact maps and assigning each genomic region to its sign[2]. Similarly, Eigenvector is calculated from the first eigenvector that corresponds to each contact frequency map, creating a vector annotated at each bin for both maps. These vectors are then compared using spearman's rank correlation. Because the components can have different signs that are arbitrarily assigned, MSE is not used for this method as it is sensitive to these signs and would result in falsely high scores when the maps are assigned opposite signs.

HiC1Dmetrics
HiC1Dmetrics[13] is a framework to extract various one-dimensional (1D) features from contact maps and has function modules for the analyses of one, two or multiple samples. The 1D metrics for comparing two samples include change in insulation score (ISC), change in contrast index (CIC), change in TAD separation score (SSC), delta of distal-to-local ratio (deltaDLR), correlation difference (CD), InterScore Change (IESC), IntraScore Change (IASC), Interaction Frequency Change (IFC), and Directional Relative Frequency (DRF). Specifically, ISC measures the average change of insulation scores between two samples, CIC measures the average change of the strength of TAD boundaries between two samples, SSC measures the average change of the degree of TAD separation between two samples, deltaDLR measures the average change of the distal-to-local interaction ratio between two samples, CD calculates the average correlation of locus-specific interaction profiles between two samples, IESC and IASC measures the change of observed over expected contact frequency within and between TADs, respectively, IFC evaluate the changes in significant interactions between samples, and DRF measures the asymmetric changes in inter-TAD interactions between samples. HiC1Dmetrics supports inputs of .hic, .cool, and dense matrix of intra-chromosomal contacts. Since HiC1Dmetrics could be easily applied to contact maps of local windows, for more unbiased comparison with our implemented methods, we extracted and compared the contact frequency matrix for each DEG window at five different resolutions between ESC and HFF Micro-C and Hi-C data. We only included ISC, CIC, SSC, deltaDLR, and CD in our analyses due to the failed implementation for other metrics at either of the five resolutions. To get a score for each metric of each window for downstream comparison, the average value of 1D metrics across the window was calculated. The parameters for ISC, CIC, SSC were set to 10240 for resolutions of 2048 bp, 4096 bp, 8192 bp, 10240 bp and 102400 for the resolution of 20480 bp. Similarly, the parameter for deltaDLR was set to 30,720 for the four smaller bin sizes and 307,200 for 20480 bp.

HiC1Dmetrics parameters:
(1) ISC: square size, p
(2) CIC: length of bins, p
(3) SSC: length of bins, p
(4) deltaDLR: local distance, p
(5) CD: Correlation method, p

HiCcompare
HiCcompare[14] is an R package that detects differences between multiple contact matrices. It generates p-values for each bin and allows for visualizing differences though mean-difference (MD) plots. For our

application, we don't use the MD plots since they are more applicable for qualitative evaluations of few map comparisons. TADcompare takes in a variety of inputs including .hic files, .mcool files, and data from HiCPro. We used .mcool files from ESC and HFF Micro-C and Hi-C data, and compared them at a chromosome-wide level. We only did this for chromosome 21 and 22 due to the tools compute and time requirements. We then subsetted the output for each window that we compared. This analysis was performed separately for different resolution datasets. For the score, we used the number of bins in each window that were significant (p-value < 0.05).

HiCcompare parameters:
(1) degree of polynomial to be used for loess, degree
(2) the amount of data used to build the loess model, span
(3) automatic span selection criterion, loess.criterion
(4) the standard deviation of the fuzzing used to produce a Hi-C matrix from your data with few true differences, SD
(5) the number of changes to add into the Hi-C matrix created, numChanges
(6) the fold change of the changes added to the Hi-C matrix, FC
(7) the alpha level for hypothesis testing, alpha
(8) fold change threshold desired to call a detected difference significant, diff.thresh
(9) number of iterations for the permutation test, iterations
(10) the threshold of average expression, where Z-scores will be set to 0 if the corresponding average expression value is less than the value, A.min
(11) whether p-value adjustment be performed on a per distance basis, adjust.dist
(12) the method for p-value adjustment, p.method

Insulation

Insulation measures the locus-specific contact frequency, thus reflecting the segmentation of the genome into domains. The profile is calculated using a diamond-shaped window-based method proposed by Crane et al.[15]. Specifically, a square (a W x W diamond-shaped window) is slid along each diagonal bin of the matrix and the averaged contact frequency within each window is calculated and called as insulation score. Bins with a low insulation score indicate a high insulatory effect, thus the bins reaching the local minima are identified as candidate TAD boundaries.

Correlation or MSE can be applied to the insulation tracks of two conditions for a scalar disruption score. The magnitude of the insulation score is dependent on differences in contact intensity between the two maps at each bin, and therefore is sensitive to global change in contact frequency. Variants in regions of DNA with wider ranges of contact intensity (high contrast) may have inflated insulation scores relative to other regions. This method can potentially be improved by adjusting the following parameters: the size of central window X, i.e. the region for which the insulation score is being calculated (default: 20kb).

Insulation parameters:
(1) size of central window (region where insulation score is calculated, window_size)

Loops

Chromatin loops are the positions where a pair of loci showing closer proximity compared to loci lying between them, corresponding to pixels with higher contact frequency than the ones in their neighborhood. We identify loops by comparing regions with their local background, as in HiCCUPS[8]. Specifically, for

each bin in the upper triangle window of the matrix, we first check whether it is a local maximum (across neighborhood window size *w*) and then calculate the mean signal of center window (window size *p*) surrounding the bin as well as the mean signal in a donut-shape neighborhood, a lower-left neighborhood, vertical and horizontal neighborhoods around the pixel. The bins enriched above its neighborhood with ratios of mean signals of the center window to the neighborhoods higher than certain thresholds are considered as candidate loops. The bins at the corners are not considered. Loops that are the same, gained, lost between two Hi-C matrices are identified. The loops that are located within a window of size *r* of one another are treated as the same. This method can potentially be improved by adjusting the following parameters: the center window size (*p*), window size (*w*), threshold of the ratio of center window to donut and lower-left filter, threshold of the ratio of center window to vertical filter, threshold of the ratio of center window to horizontal filter, and the upper bound of bin distance where two loops are considered as same one.

Loops parameters:
(1) center window size, p
(2) window size, w
(3) threshold of the ratio of center window to donut and lower-left filter
(4) threshold of the ratio of center window to vertical filter
(5) threshold of the ratio of center window to horizontal filter
(6) upper bound of bin distance where two loops are considered as the same one.

SCC
Contact frequency in Hi-C maps is known to exhibit a distance-dependent decay. The high similarity of the dependence pattern might bias the correlation between Hi-C maps, thus causing high, spurious correlations. Stratum-adjusted Correlation Coefficient (SCC) addresses this distance-dependence effect by stratifying Hi-C data based on genomic distance, calculating a Pearson correlation coefficient for each stratum and aggregating the weighted stratum-specific correlation coefficients with weights derived from the generalized Cochran–Mantel–Haenszel (CMH) statistic[16]. SCC values range from -1 to 1 and share a similar interpretation as standard correlations. The equation to calculate SCC can be written as:

$$\rho_S = \sum_k w_k p_k$$

SCC was first implemented for Hi-C map comparison by Yang et al. in the R package HiCRep [16]. By including distance-aware weights, SCC is able to measure the overall reproducibility of the Hi-C matrices better than standard correlations and is resistant to decreased resolution. However, SCC is less likely to identify small changes in TAD substructures compared to some other methods surveyed here (see **Table 1**).

Selfish
Selfish[17] is a tool to identify differential chromatin interactions between two contact maps based on self-similarity metric. The idea is that each interaction frequency could be represented by its adjacent interactions. Specifically, a contact map is first convoluted with a set of Gaussian filters with different radii, resulting in a feature vector. Then, the difference between the first-order derivatives of feature vectors is calculated to indicate the significance of a change between interaction frequencies across different contact maps. Selfish supports multiple input formats, including plain text, .hic, .cool and HiC-Pro style of bed-matrix pairs, and has the MATLAB and python implementations. We used the python version on the ESC and HFF Micro-C and Hi-C data at a chromosome-wide level, followed by subsetting the number of differential chromatin interactions (q-value <0.05) between ESC and HFF in each DEG window on chromosome 21 and 22. Same as other methods, the analyses were performed at five different resolutions.

Selfish parameters:
(1) sparsity threshold used to filter out differential interactions falling in sparse regions, st
(2) do not use sparsity checking, ns
(3) maximum distance between interacting loci, d
(4) q-value threshold for which the results will be written to the TSV file, t
(5) iteration count, i
(6) include the interactions that are zero in one of the contact maps, nm.

TADcompare
TADcompare[18] is an R package that identifies differential TADs between multiple contact matrices. It provides three functions, TADcompare for finding differential TADs, TimeCompare for performing time course analysis, and ConsensusTADs for identifying consensus boundaries. For our application, TADcompare is the only applicable function since it can compare two different maps. TADcompare identifies differential TAD boundaries between two samples by leveraging the eigenvector gap, which quantifies the degree of change in contact patterns between interacting regions, therefore measuring the possibility of a locus as a boundary. The boundary difference between samples is evaluated through a z-score, which is calculated from the differences of the eigenvector gap that closely adheres to a log-normal distribution. TADcompare takes in a variety of inputs including non-normalized interaction matrices, .hic files, .mcool files, and pre-specified TADs as an additional input. We used .mcool files from ESC and HFF Micro-C and Hi-C data, and compared them at a chromosome-wide level. We only did this for chromosome 21 and 22 due to the tools computing and time requirements. We then subsetted the output for each window that we compared. This analysis was performed separately for different resolution datasets. The resolution parameter was not specified since it can be estimated automatically. Results should be consistent regardless of window size so that parameter was not specified either. In total, default values were used for all the parameters. For the score, we used the number of TADs in each window that were not Non-differential (e.g. Differential or Shifted) from the TAD_Frame output.

TADcompare parameters:
(1) threshold for differential boundary score, z_thresh
(2) size of sliding window for TAD detection (measured in bins), window_size
(3) percent of non-zero interaction frequencies for a given bin to be included in the analysis, gap_thresh

TADs
TAD boundaries are called by finding the local minima of the insulation profile. The boundary strength is calculated for each local minima using peak prominence and candidates with strength above a threshold are referred to as TAD boundaries[19]. The scores for the bins at the end of the diagonal and within the window size are not calculated. The overlap, gain, and loss of TAD boundaries between two Hi-C matrices are reported to show their consistency and changes. The boundary locations within a set resolution r are considered the same. This method could be further improved by changing the following parameters: window size (w), threshold of boundary strength, and upper bound of distance when two TAD boundaries are considered the one.

TADs parameters:
(1) window size, w
(2) threshold of boundary strength
(3) upper bound of distance when two TAD boundaries are considered one

Triangle
Global methods (Correlation, MSE) ignore the physical relationships between pixels of the map when they are flattened into vectors. They are therefore over-simplified characterizations of the relationships between maps. This method tries to leverage our understanding that contacts are represented by different subsets of triangles within the larger map to address this gap. The triangle-based method compares the average contact intensity within all sub-triangles of two contact maps. To do so, sub-triangles with base corresponding to n bins, for n between 2 and 447, are averaged. This results in a flattened representation of the map with the average contact intensity of all sub-triangles instead of just each pixel on its own. The flattened representations can then be compared with either MSE or correlation.

The performance of this method depends on the correlation or MSE used over the sub-triangles (see their individual pros and cons). The advantage over those global methods is that triangle comparison is more feature-informed to capture relevant contact relationships. Because there are so many more smaller triangles than larger triangles, this method likely prioritizes more local changes; however, one could weight the triangles or subset to only the larger or smaller sub-triangles to prioritize only larger or smaller scale interactions. One caveat is that this method is significantly slower than other methods, but speed can be improved by creating lower resolution maps before computing.

Predicted contact map comparisons

We simultaneously clustered the perturbed map pairs and scores across methods to identify groups of perturbations that differentiate them (**Supplementary Fig. 5c**). Some methods, such as insulation (corr), SSIM, and DI (corr), produce scores which are more uniformly distributed and less extreme across perturbations, highlighting the necessity of appropriate normalization when comparing across methods (**Supplementary Fig. 5d i and vi**). We also find that perturbations created by CTCF insertion group together, as they are often the most disruptive of 3D organization. However, we observed substantial sub-structure within the cluster, reflecting differences in the behavior of scores on these maps. For example, cluster ***i*** is highly scored by all methods, and a representative perturbation example shows a variety of changes: gained loops, lost stripes, and boundary changes. The magnitude of changes in this set likely contributes to the universally high scores. Clusters ***iv*** and ***v*** are primarily composed of CTCF insertions, where scores are similar across most methods, but higher only for MSE-based methods. Profile i***v*** is the most dissimilar. Here, the representative map pair has minimal structural differences but extreme contrast, suggesting that this cluster is defined by examples of high dynamic range that are over-prioritized by MSE-based methods (**Supplementary Fig. 5d iv**).

Supplementary References

1. Dixon, J. R. *et al.* Chromatin architecture reorganization during stem cell differentiation. *Nature* **518**, 331–336 (2015).
2. Lieberman-Aiden, E. *et al.* Comprehensive mapping of long-range interactions reveals folding principles of the human genome. *Science* **326**, 289–293 (2009).
3. Fudenberg, G., Kelley, D. R. & Pollard, K. S. Predicting 3D genome folding from DNA sequence with Akita. *Nat. Methods* **17**, 1111–1117 (2020).
4. Tan, J. *et al.* Cell-type-specific prediction of 3D chromatin organization enables high-throughput in silico genetic screening. *Nat. Biotechnol.* (2023) doi:10.1038/s41587-022-01612-8.
5. Schwessinger, R. *et al.* DeepC: predicting 3D genome folding using megabase-scale transfer learning. *Nat. Methods* **17**, 1118–1124 (2020).
6. Yang, R., Das, A., Gao, V. R., Karbalayghareh, A. & Noble, W. S. Epiphany: predicting hi-c contact maps from 1d epigenomic signals. *bioRxiv* (2021).
7. Galan, S. *et al.* CHESS enables quantitative comparison of chromatin contact data and automatic feature extraction. *Nat. Genet.* **52**, 1247–1255 (2020).
8. Rao, S. S. P. *et al.* A 3D map of the human genome at kilobase resolution reveals principles of chromatin looping. *Cell* **159**, 1665–1680 (2014).
9. Dixon, J. R. *et al.* Topological domains in mammalian genomes identified by analysis of chromatin interactions. *Nature* **485**, 376–380 (2012).
10. Chakraborty, A., Wang, J. G. & Ay, F. dcHiC detects differential compartments across multiple Hi-C datasets. *Nat. Commun.* **13**, 6827 (2022).
11. Nagano, T. *et al.* Cell-cycle dynamics of chromosomal organization at single-cell resolution. *Nature* **547**, 61–67 (2017).
12. Zhou, J. *et al.* Robust single-cell Hi-C clustering by convolution- and random-walk–based imputation. *Proceedings of the National Academy of Sciences* **116**, 14011–14018 (2019).

13. Wang, J. & Nakato, R. HiC1Dmetrics: framework to extract various one-dimensional features from chromosome structure data. *Brief. Bioinform.* **23**, (2022).

14. Stansfield, J. C., Cresswell, K. G., Vladimirov, V. I. & Dozmorov, M. G. HiCcompare: an R-package for joint normalization and comparison of HI-C datasets. *BMC Bioinformatics* **19**, 279 (2018).

15. Crane, E. *et al.* Condensin-driven remodelling of X chromosome topology during dosage compensation. *Nature* **523**, 240–244 (2015).

16. Yang, T. *et al.* HiCRep: assessing the reproducibility of Hi-C data using a stratum-adjusted correlation coefficient. *Genome Res.* **27**, 1939–1949 (2017).

17. Pindyurin, A. V. & Fishman, V. *The Role of High-Order Chromatin Organization in Gene Regulation*. (Frontiers Media SA, 2022).

18. Cresswell, K. G. & Dozmorov, M. G. TADCompare: An R package for differential and temporal analysis of topologically associated domains. *Front. Genet.* **11**, 158 (2020).

19. Open2C *et al.* Cooltools: enabling high-resolution Hi-C analysis in Python. *bioRxiv* 2022.10.31.514564 (2022) doi:10.1101/2022.10.31.514564.
