# Supplementary material for: Comparing chromatin contact maps at scale: methods and insights
Source: Nat Methods. 2025 Mar 19;22(4):824–33. doi: 10.1038/s41592-025-02630-5 (PMC11978506; doi:10.1038/s41592-025-02630-5)
Supplement: Supplementary file 2 — Reporting Summary [file 41592_2025_2630_MOESM2_ESM.pdf]

nature portfolio

Corresponding author(s): John A. Capra, Katherine S. Pollard

Last updated by author(s): Nov 8, 2024

Reporting Summary

Nature Portfolio wishes to improve the reproducibility of the work that we publish. This form provides structure for consistency and transparency in reporting. For further information on Nature Portfolio policies, see our Editorial Policies and the Editorial Policy Checklist.

Statistics

For all statistical analyses, confirm that the following items are present in the figure legend, table legend, main text, or Methods section.

n/a	Confirmed	
☐	☒	The exact sample size (*n*) for each experimental group/condition, given as a discrete number and unit of measurement
☒	☐	A statement on whether measurements were taken from distinct samples or whether the same sample was measured repeatedly
☐	☒	The statistical test(s) used AND whether they are one- or two-sided<br>*Only common tests should be described solely by name; describe more complex techniques in the Methods section.*
☒	☐	A description of all covariates tested
☒	☐	A description of any assumptions or corrections, such as tests of normality and adjustment for multiple comparisons
☐	☒	A full description of the statistical parameters including central tendency (e.g. means) or other basic estimates (e.g. regression coefficient) AND variation (e.g. standard deviation) or associated estimates of uncertainty (e.g. confidence intervals)
☐	☒	For null hypothesis testing, the test statistic (e.g. *F*, *t*, *r*) with confidence intervals, effect sizes, degrees of freedom and *P* value noted<br>*Give P values as exact values whenever suitable.*
☒	☐	For Bayesian analysis, information on the choice of priors and Markov chain Monte Carlo settings
☒	☐	For hierarchical and complex designs, identification of the appropriate level for tests and full reporting of outcomes
☐	☒	Estimates of effect sizes (e.g. Cohen's *d*, Pearson's *r*), indicating how they were calculated

Our web collection on statistics for biologists contains articles on many of the points above.

Software and code

Policy information about availability of computer code

Data collection	No software was used for data collection.
Data analysis	python packages: Python v3.10.12, cooler v0.9.2, cooltools v0.5.4, Matplotlib v3.7.2, Numpy v1.23.5, Pandas v1.5.3, scipy v1.10.1, Seaborn v0.12.2, h5py v3.8.0, hicrep v0.2.6, sklearn v1.0.2, skimage v0.19.3, Arrowhead of Juicer Tools v1.8.9, CHESS v0.3.8, HiC1Dmetrics v0.2.5, Selfish v1.14.0.<br>R packages: Rstudio v0.16.0, HiCcompare v1.26.0, TADcompare v1.14.0, dcHiC v1.<br>conda v4.12.0.<br>Other: Akita v1.<br>All original code and resulting data for experimental and in silico scored contact map pairs are available at https://github.com/pollardlab/contact_map_scoring.

For manuscripts utilizing custom algorithms or software that are central to the research but not yet described in published literature, software must be made available to editors and reviewers. We strongly encourage code deposition in a community repository (e.g. GitHub). See the Nature Portfolio guidelines for submitting code & software for further information.

Data

Policy information about availability of data

All manuscripts must include a data availability statement. This statement should provide the following information, where applicable:

- Accession codes, unique identifiers, or web links for publicly available datasets
- A description of any restrictions on data availability
- For clinical datasets or third party data, please ensure that the statement adheres to our policy

Micro-C and Hi-C datasets used in map comparisons and RNA-seq datasets for identifying DEG genes are publicly available from 4DN data portal (Micro-C for ESC and HFF: 4DNES21D8SP8 and 4DNESWST3UBH, Hi-C for ESC and HFF: 4DNESX75DD7R and 4DNESNMAAN97, and RNA-seq for ESC and HFF: 4DNES3IOYG74 and 4DNESFH3EHTU). The reference genome from the hg38 build was used.

Resulting data for experimental and in silico scored contact map pairs have been deposited at https://github.com/pollardlab/contact_map_scoring.

Human research participants

Policy information about studies involving human research participants and Sex and Gender in Research.

Reporting on sex and gender	N/A
Population characteristics	N/A
Recruitment	N/A
Ethics oversight	N/A

Note that full information on the approval of the study protocol must also be provided in the manuscript.

Field-specific reporting

Please select the one below that is the best fit for your research. If you are not sure, read the appropriate sections before making your selection.

☒ Life sciences ☐ Behavioural & social sciences ☐ Ecological, evolutionary & environmental sciences

For a reference copy of the document with all sections, see nature.com/documents/nr-reporting-summary-flat.pdf

Life sciences study design

All studies must disclose on these points even when the disclosure is negative.

Sample size	Results are based on a sample size of 256 1-megabase regions of human chromosomes 21 and 22. We chose these two chromosomes since they are the smallest chromosomes, and some of the methods we evaluated have computational limitations and would not run on more data. After quality control filtering, this results in a sample size of 256 regions for comparison.
Data exclusions	Contact maps that were missing at least 60% of the information were excluded from the study.
Replication	Our benchmark results focus on differences between methods on the same input data. The benchmark includes data from the same genome region across biological replicates (different cell lines) and technical replicates (different Hi-C experiments). We did not include any other forms of replication experiments.
Randomization	There are no experimental groups in this study.
Blinding	There was no group allocation for this study.

Reporting for specific materials, systems and methods

We require information from authors about some types of materials, experimental systems and methods used in many studies. Here, indicate whether each material, system or method listed is relevant to your study. If you are not sure if a list item applies to your research, read the appropriate section before selecting a response.

Materials & experimental systems

n/a	Involved in the study
☒	☐ Antibodies
☒	☐ Eukaryotic cell lines
☒	☐ Palaeontology and archaeology
☒	☐ Animals and other organisms
☒	☐ Clinical data
☒	☐ Dual use research of concern

Methods

n/a	Involved in the study
☒	☐ ChIP-seq
☒	☐ Flow cytometry
☒	☐ MRI-based neuroimaging
